# Validation of PET/MRI attenuation correction methodology in the study of brain tumours

**DOI:** 10.1186/s12880-020-00526-8

**Published:** 2020-11-25

**Authors:** Francesca De Luca, Martin Bolin, Lennart Blomqvist, Cecilia Wassberg, Heather Martin, Anna Falk Delgado

**Affiliations:** 1 Department of Clinical Neuroscience, Karolinska Institutet, Stockholm, Sweden; 2 Department of Neuroradiology, Karolinska University Hospital, Stockholm, Sweden; 3 Department of Molecular Medicine and Surger, Karolinska Institutet, Stockholm, Sweden; 4 Department of Medical Radiation Physics and Nuclear Medicine, Karolinska University Hospital, Stockholm, Sweden

**Keywords:** Attenuation correction, PET/MRI, ZTE–AC, Atlas-AC, CT-AC, Brain tumour, Tumour recurrence, Magnetic resonance imaging, Biomarkers, Prognosis

## Abstract

**Background:**

This study aims to compare proton density weighted magnetic resonance imaging (MRI) zero echo time (ZTE) and head atlas attenuation correction (AC) to the reference standard computed tomography (CT) based AC for ^11^C-methionine positron emission tomography (PET)/MRI.

**Methods:**

A retrospective cohort of 14 patients with suspected or confirmed brain tumour and ^11^C-Methionine PET/MRI was included in the study. For each scan, three AC maps were generated: ZTE–AC, atlas-AC and reference standard CT-AC. Maximum and mean standardised uptake values (SUV) were measured in the hotspot, mirror region and frontal cortex. In postoperative patients (n = 8), SUV values were additionally obtained adjacent to the metal implant and mirror region. Standardised uptake ratios (SUR) hotspot/mirror, hotspot/cortex and metal/mirror were then calculated and analysed with Bland–Altman, Pearson correlation and intraclass correlation reliability in the overall group and subgroups.

**Results:**

ZTE–AC demonstrated narrower SD and 95% CI (Bland–Altman) than atlas-AC in the hotspot analysis for all groups (ZTE overall ≤ 2.84, − 1.41 to 1.70; metal ≤ 1.67, − 3.00 to 2.20; non-metal ≤ 3.04, − 0.96 to 3.38; Atlas overall ≤ 4.56, − 1.05 to 3.83; metal ≤ 3.87, − 3.81 to 4.64; non-metal ≤ 4.90, − 1.68 to 5.86). The mean bias for both ZTE–AC and atlas-AC was ≤ 2.4% compared to CT-AC. In the metal region analysis, ZTE–AC demonstrated a narrower mean bias range—closer to zero—and narrower SD and 95% CI (ZTE 0.21–0.48, ≤ 2.50, − 1.70 to 2.57; Atlas 0.56–1.54, ≤ 4.01, − 1.81 to 4.89). The mean bias for both ZTE–AC and atlas-AC was within 1.6%. A perfect correlation (Pearson correlation) was found for both ZTE–AC and atlas-AC compared to CT-AC in the hotspot and metal analysis (ZTE ρ 1.00, p < 0.0001; atlas ρ 1.00, p < 0.0001). An almost perfect intraclass correlation coefficient for absolute agreement was found between Atlas-, ZTE and CT maps for maxSUR and meanSUR values in all the analyses (ICC > 0.99).

**Conclusions:**

Both ZTE and atlas-AC showed a good performance against CT-AC in patients with brain tumour.

## Background

In neuro-oncology, positron emission tomography/magnetic resonance imaging (PET/MRI) combines Positron Emission Tomography (PET) functional metabolic information with the magnetic resonance imaging (MRI) morphological appearance, allowing simultaneous data acquisition with the potential to overcome the intrinsic limitations of MRI [1]. Among the existing radiopharmaceuticals used in PET imaging, ^11^C-methionine (MET) is considered one of the most suitable amino acid tracers for brain tumours [2]. The uptake of MET by transmembrane transport via sodium-independent L-transporters reflects the concentration gradient and cellular proliferation related to protein synthesis within the tumour. The distribution of ^11^C-methionine has potential to characterise primary brain tumour/metastases, assess the efficacy of oncological treatment and differentiate radionecrosis from tumour recurrence [3–5]. Due to these characteristics, ^11^C-methionine provides a higher selectivity for the identification of brain gliomas, especially for hypo- or isometabolic lesions on ^18^F-Flurodeoxyglucose (FDG) PET [6, 7] and a diagnostic accuracy similar to ^18^F-fluoroethyl-l-tyrosine (FET) [8]. For accurate quantification of the radioactivity concentration measured by PET, correction for photon attenuation is needed. Conversion of the MRI signal into attenuation values is challenging, hampering accurate AC in PET/MRI [9]. Several methods aiming to improve vendor specific MR-AC have been described [10–12]. Nonetheless, head CT scan is still needed for accurate AC, leaving PET/MRI dependent upon a separate CT and additional radiation exposure [13–15]. In comparison to PET/CT, PET/MRI lacks a clinically accepted standard method to directly obtain AC maps.

The unsolved AC issues in PET/MRI relate to the fact that proton density in MRI does not directly correlate to radiodensity [16, 17]. PET/MRI AC is especially challenging close to bony structures [18], air and metal implants [19]. Metal implants are encountered in postoperative brain tumour patients, introducing risk for errors in the AC near the postoperative site. Several attempts to improve MR-AC close to metal in PET/MRI has been made, for example using time-of-flight (TOF) [20, 21].

A recently developed template-based method, Zero Echo Time (ZTE), for AC in PET/MRI has been developed and tested in non-surgical patients, providing an accurate AC map when compared to attenuation correction with PET emission scan [10, 18, 22, 23]. The aim of this current study is to further evaluate ZTE–AC in pre- and postoperative patients with suspected or confirmed brain tumour for correct SUV quantification in the presence of surgical metal implants and clinical use of PET/MRI study of brain tumours. Correct AC in PET/MRI will improve diagnostic accuracy and guide treatment-based decisions in clinical practice.

To our knowledge, this is the first study evaluating ZTE–AC and atlas-AC compared to the reference standard CT-AC for brain MET PET/MRI.

## Methods

The aim of the study is to retrospectively investigate ZTE–AC and atlas-AC compared to the reference standard CT-AC for MET PET/MRI in pre- and postoperative patients with suspected or confirmed brain tumour. Maximum and mean standardised uptake values (SUV) were measured in the hotspot, mirror region and frontal cortex. In postoperative patients (n = 8), SUV values were additionally obtained adjacent to the metal implant and mirror region.

### Subjects

We evaluated 18 lesions from 14 consecutive patients (6 male, 8 female, median age 43 years, range 29–73) who had acquired MET PET/CT (not analysed further in this article) and 3 T PET/MRI (GE Healthcare, Waukesha, WI) at Karolinska University Hospital from January to June 2019. Inclusion criteria were ^11^C-MET PET/MRI acquisition, availability of atlas-AC map, proton density weighted ZTE–AC map and CT-based AC map as reference standard and suspected or confirmed brain tumour. The study was approved by the Swedish Ethical Review Authority (2019–01309), through which informed consent was waived.

### Data acquisition and image reconstruction

#### Acquisition of PET/MRI

First, a MET PET/CT was acquired per clinical protocol 15 min after injection of 4 mBq/kg (max 400 mBq) ^11^C-methionine (MET), with an acquisition time of 15 min. A low dose CT (7.5 mAs 120 kVp) was acquired for both clinical PET/CT AC and PET/MRI CT-AC. After the standard PET/CT exam, subjects were moved to the PET/MRI facility, located in an adjacent building, and scanned in a GE Signa 3 T PET/MRI scanner using a 24-channel head-neck unit coil (GE Healthcare, Waukesha, WI). The average time from MET injection to PET acquisition at the PET/MRI scanner was 59 min (41–81), depending on the mobility of the subject. The mean duration of the MET-PET exam at the PET/MRI was 17.1 min (range 16–30 min). The MRI acquisition included the following scan protocol for AC: a 3D Liver Acquisition with Volume Acceleration-flexible (LAVA Flex) T1-weighted (GE Healthcare, Waukesha, WI) and a ZTE sequence were acquired. The LAVA Flex sequence was acquired for 14 s with 1 Number of excitations (NEX), 500 mm field of view (FOV), a 256 × 256 matrix and a 5.2 mm slice thickness and was used to generate water, fat and in- and out of phase echoes. ZTE sequence acquisition time was 42 s with 4 NEX, 264 mm FOV, a 110 × 110 matrix and 2.4 mm slice thickness. The clinical MRI protocol included T1, T2, T2*, postcontrast T1, DWI, ADC and Fractional Anisotropy for all the patients. Additional perfusion (ID 5–16) and spectroscopy (ID 10, 12, 14–18) sequences were also acquired.

#### Attenuation correction maps

For each subject, two different MR-based AC maps (ZTE–AC and atlas-AC) were generated and used for PET reconstruction and SUV calculation. For comparison, CT-AC was used as a reference standard method for PET AC.

#### ZTE based attenuation correction map

Following the method described in Wiesinger et al. [24] and using the MATLAB (version R2018b; The MathWorks) PET toolbox, a ZTE-based AC map was generated using:

- Histogram-based bias correction and normalization
- Tissue classification using a simple thresholding technique for soft tissue/bone and bone/air
- Attenuating for bone (300–2000 HU) with a linear correlation between CT and ZTE MRI values
- Classification of soft tissue fixed at 42 HU

#### Atlas-based attenuation correction map

Following the method described in Wollenweber et al. [25], an atlas-based AC map was generated using:

- Enhancement of bone in the 3D FSPGR T1-weighted images using a Hessian filter
- Rigid and non-rigid registration of bone-enhanced MRI to the CT skull atlas (provided by the manufacturer), creating a pseudo-CT
- Transformation of the pseudo-CT to an AC map for 511 keV photons through standard energy conversion and resampling
- Head coil and bed added to the AC map using a template

#### CT-based attenuation correction map

Following the method described in Burger et al. [26], CT-based AC map was generated using:

- Rigid registration between the low dose head CT (7.5 mAs 120 kVp) and ZTE MR images using FMRIB’s Linear Image Registration (FLIRT) Tool Software Library v6.0, Oxford, UK. and in-house created MATLAB script
- CT transformation following the same procedure as in the atlas-AC map

For each AC map, PET images were reconstructed using the GE PET toolbox (version MP26, GE Healthcare, Waukesha, WI) using TOF ordered subsets expectation maximization (OSEM) with three iterations, 16 subsets, and a 128 × 128 reconstruction matrix with a 256 mm FOV. A 3 mm Gaussian post-filter was applied to the reconstructed images.

### ROI analysis

**Fig. 1:**
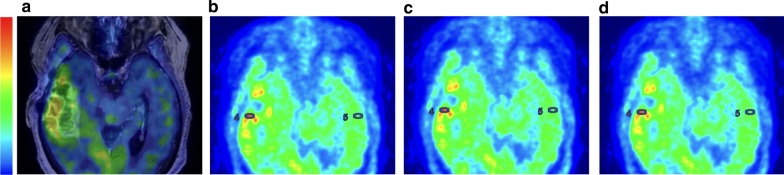
Hotspot analysis. MET PET/MRI T1-weighted contrast for a representative brain tumour patient with metal implants (**a**). SUV hotspot and SUV mirror in CT-AC (**b**), atlas-AC (**c**) and ZTE–AC (**d**), demonstrating differences between the three AC methods

After MET PET/MRI acquisition, one of the authors (FDL) analysed the PET images using AW Server Volume Viewer 3D Viewer PET (GE Healthcare, Waukesha, WI). The analysis was blinded to the neuropathological diagnosis and clinical information. Results were analysed for the overall group and two subgroups (non-metal and metal), including pre- and postoperative patients respectively with suspected or confirmed brain tumour. Standardised uptake value (SUV) maps were calculated, normalized to body weight. A 27.4 mm^2^ elliptical region of interest (ROI) was delineated to include the PET hotspot with the help of gadolinium-enhanced T1weighted images from the PET/MRI or the prior MRI (Fig. 1a–d). For standardised uptake ratio (SUR) analysis, two different background regions were delineated:

- Elliptic ROI (27.4 mm^2^) in the hotspot mirror region
- Free-hand 2D ROI in the contralateral frontal cortex at the level of the basal ganglia

**Fig. 2:**
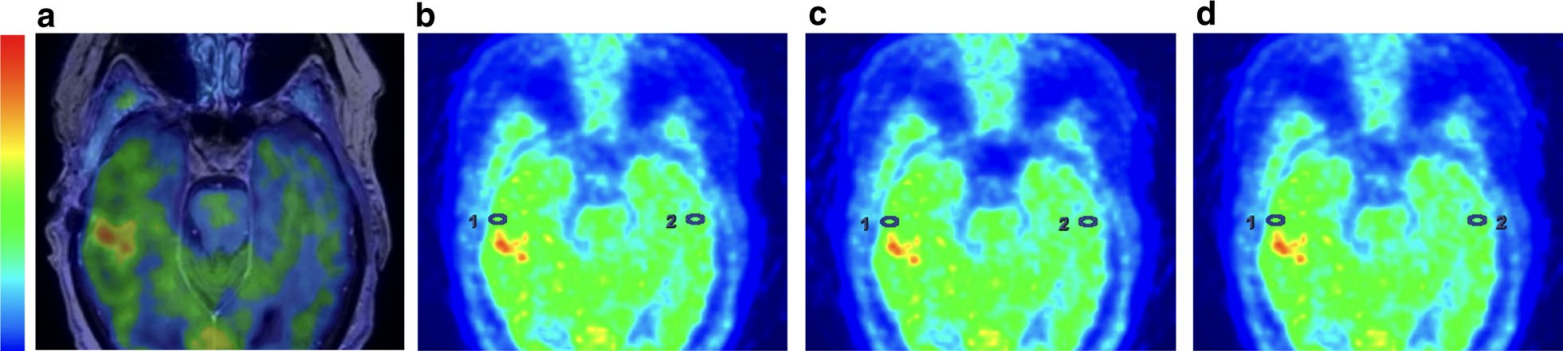
Metal analysis. MET PET/MRI T1-weighted contrast for a representative brain tumour patient with metal implants (**a**). SUV adjacent to the metal and mirror region in CT-AC (**b**), atlas-AC (**c**) and ZTE–AC (**d**), demonstrating differences between the three AC methods

In the postoperative patients, all presenting with metal implants, SUV values were additionally obtained in the parenchyma tangential to the longitudinal axis of the adjacent metal implant and mirror region (Fig. 2a–d). In subjects with multiple surgical implants, the metal implant closest to the postoperative cavity and/or lesion was selected. SUR values – maxSURhotspot/mirror and maxSURhotspot/cortex; meanSURhotspot/mirror and meanSURhotspot/cortex; maxSURmetal/mirror and meanSURmetal/mirror—were calculated.

### Statistical analysis

SUR values were analysed with the Bland–Altman (mean bias, SD, 95% CI), the Pearson correlation test (p < 0.05) and intraclass correlation reliability (ICC > 0.99) for absolute agreement.

Bland–Altman and Pearson correlation tests were performed using GraphPad Prism 8.4.3 (GraphPad Software, San Diego, California). Intraclass correlation reliability was performed using Statistical Package for Social Sciences (SPSS, IBM Corp. Released 2019. Version 26.0. Armonk, NY: IBM Corp.).

Mean bias was calculated as follows [27]:

$$Bias \% (100*\left( \frac{ZTE/ATLAS-CT}{CT}\right) )$$

Outlier analysis was performed using Robust regression and Outlier removal (ROUT) test [28].

## Results

Fourteen patients with 18 lesions were included in the study. Seventeen lesions had a hotspot at PET/MRI and the diagnoses included 6 gliomas (WHO grade IV, n = 1; grade III, n = 3; grade II, n = 2), 9 metastases (melanoma, n = 3; breast, n = 4; kidney, n = 1; lung, n = 1) per WHO classification (2, 3) and two suspected brain tumours (two suspected low grade astrocytomas). The non-metal subgroup included 10 preoperative lesions. Seven out of seventeen lesions occurred in postoperative patients with metal implant. In the metal analysis (n = 8), one additional postoperative patient with astrocytoma WHO grade II without a hotspot on PET/MRI was included [29, 30]. The majority of the metal implants involved CranioFix titanium craniotomy clamps (B. Braun Medical, Melsungen, Germany) or Low-profile titanium plates or screws (DePuy Synthes, Warsaw, Indiana, United States). The majority of the lesions (n = 16) were treated using currently available treatment options in neuro-oncology and/or: surgery, chemotherapy, gamma knife radiosurgery or radiotherapy. Table 1.

**Table 1:** Baseline demographic and patients’ characteristics

Characteristics of patients with ^11^C-MET PET/MRI Patients n = 14 ^11^C-MET PET/MRI scans n = 15 Lesions n = 18							
	Neuropathological confirmation (Yes/No)	Pathological diagnosis	Metal implant and analysis (Yes/No)	Treatment (a/o C, GNR, R, S) (Yes/No)	Hotspot analysis overall group (Yes/No)	Hotspot analysis metal subgroup (Yes/No)	Hotspot analysis non-metal subgroup (Yes/No)
ID1	Yes	Melanoma metastasis	No	Yes	Yes	No	Yes
ID2	Yes	Astrocytoma WHO II	Yes	Yes	Yes	Yes	No
ID3	Yes	Anaplastic Astrocytoma WHO III	Yes	Yes	Yes	Yes	No
ID4	Yes	Lung cancer metastasis	No	Yes	Yes	No	Yes
ID5	Yes	Melanoma metastasis	Yes	Yes	Yes	Yes	No
ID6	Yes	BC metastasis	No	Yes	Yes	No	Yes
ID7	Yes	BC metastasis	No	Yes	Yes	No	Yes
ID8	Yes	BC metastasis	No	Yes	Yes	No	Yes
ID9	Yes	BC metastasis	No	Yes	Yes	No	Yes
ID10	Yes	Kidney cancer metastasis	No	Yes	Yes	No	Yes
ID11	Yes	OD WHO II-III	No	Yes	Yes	No	Yes
ID12	No	–	No	No	Yes	No	Yes
ID13	Yes	GBM WHO IV	Yes	Yes	Yes	Yes	No
ID14	Yes	Astrocytoma WHO II	Yes	Yes	No	No	No
ID15	Yes	OD WHO II	Yes	Yes	Yes	Yes	No
ID16 (same as in ID5)	Yes	Melanoma metastasis	Yes	Yes	Yes	Yes	No
ID17	No	–	No	No	Yes	No	Yes
ID18	Yes	Astrocytoma WHO II-III	Yes	Yes	Yes	Yes	No

*BC* breast cancer; *C* chemotherapy, *GB* glioblastoma, *GNR* gamma knife radiosurgery, *R* radiotherapy, *OD* oligodendroglioma, *WHO* World Health Organization

### Hotspot analysis

**Fig. 3:**
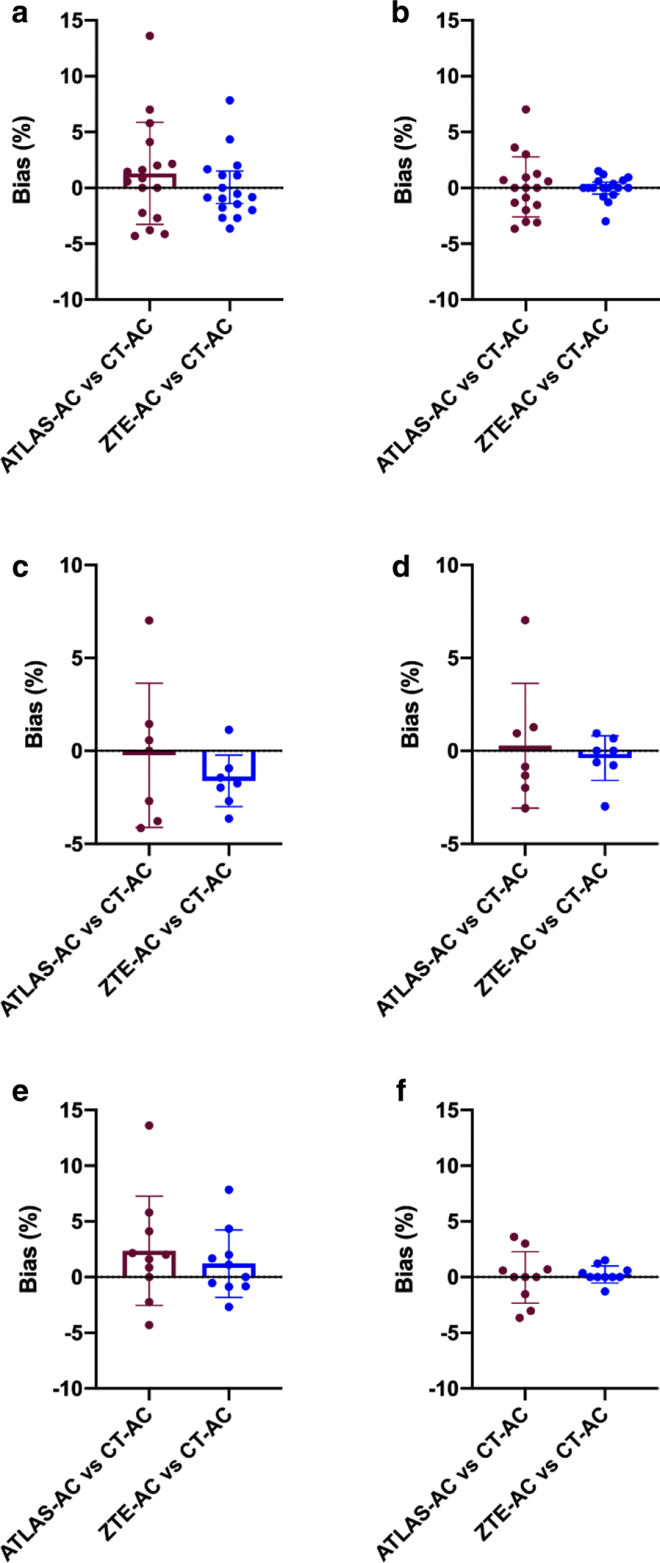
Bland Altman for SURhotspot/cortex and SURhotspot/mirror analysis. Mean bias for maxSURhotspot/cortex (left) and maxSURhotspot/mirror (right) respectively in the overall (**a**, **b**), metal (**c**, **d**) and non-metal (**e**, **f**) subgroup. ZTE–AC and atlas-AC compared to reference standard CT-AC. Bars and whiskers are mean ± SD. Despite atlas-AC showed narrower mean bias range -closer to zero- in the metal subgroup, ZTE–AC presented narrower SD and 95% CI for all the three groups, metal subgroup included. No notable difference in AC compared to CT-AC was found for both methods

ZTE–AC demonstrated narrower SD and 95% CI than Atlas-AC in the hotspot analysis for all groups (ZTE overall ≤ 2.84, − 1.41 to 1.70; metal ≤ 1.67, − 3.00 to 2.20; non-metal ≤ 3.04, − 0.96 to 3.38; Atlas overall ≤ 4.56, − 1.05 to 3.83; metal ≤ 3.87, − 3.81 to 4.64; non-metal ≤ 4.90, − 1.68 to 5.86). Mean bias was within 2.4% for both ZTE–AC and atlas-AC compared to CT-AC. In the subgroup of patients with metal implants, atlas-AC demonstrated a narrower mean bias range—closer to zero—in three out of four analyses (atlas − 0.23 to 1.63, ZTE − 1.80 to 0.66). Results for the analysis of maxSURhotspot/cortex and maxSURhotspot/mirror are presented in Fig. 3a–f. A perfect correlation was found for both ZTE–AC and atlas-AC compared to CT-AC in the hotspot analysis (ZTE ρ 1.00, p < 0.0001; atlas ρ 1.00, p < 0.0001). Higher absolute values of correlation were found when the mirror region was chosen as background. The ROUT outliers test was performed and no outliers were identified for the analyses. Intraclass correlation reliability using absolute agreement between Atlas-, ZTE and CT maps for maxSUR and meanSUR values (hotspot/cortex; hotspot/mirror) was conducted and showed an almost perfect intraclass correlation coefficient (ICC > 0.99) in all the analyses. Detailed results for Bland–Altman, Pearson correlation and intraclass correlation reliability can be found in Additional file 1: Table S1, S3, Fig. S1–S3.

### Metal analysis

**Fig. 4:**
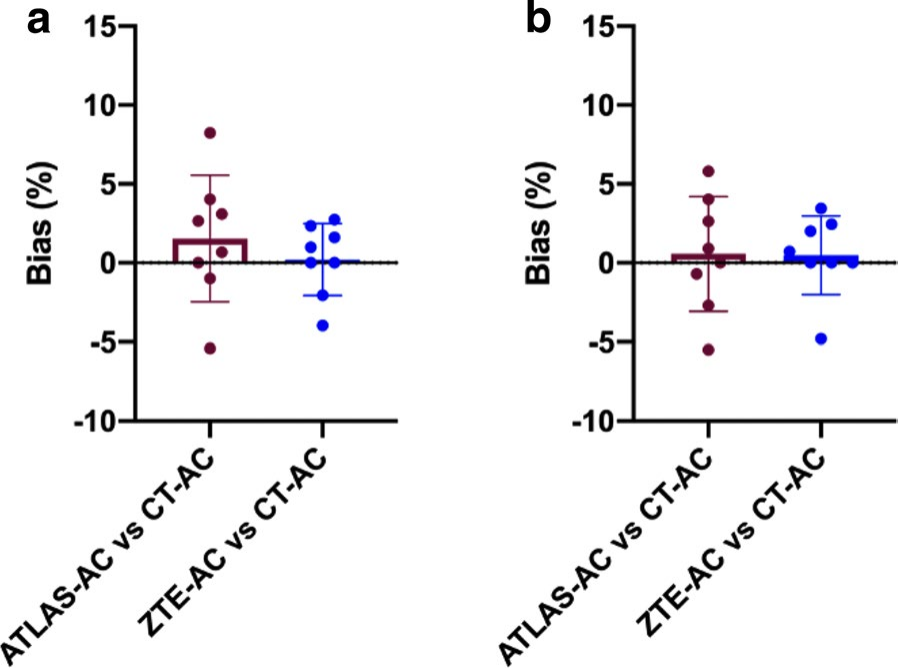
Bland Altman for SURmetal/mirror analysis. Mean bias for maxSURmetal/mirror (**a**) and meanSURmetal/mirror (**b**) in postoperative patients presenting with metal implants. ZTE–AC and atlas-AC compared to reference standard CT-AC. Bars and whiskers are mean ± SD

In the metal region analysis, ZTE–AC demonstrated a narrower mean bias range—closer to zero—SD and 95% CI (ZTE 0.21–0.48, ≤ 2.50, − 1.70 to 2.57; Atlas 0.56–1.54, ≤ 4.01, − 1.81 to 4.89) (Fig. 4a, b). Both ZTE–AC and atlas-AC showed a perfect correlation compared to CT-AC (ZTE ρ 1.00, p < 0.0001; atlas ρ 1.00, p < 0.0001). ROUT outliers test was performed and no outliers were identified for the analysis. Intraclass correlation reliability using absolute agreement between Atlas-, ZTE and CT maps for maxSUR and meanSUR values (metal/mirror) was conducted and showed an almost perfect intraclass correlation coefficient (ICC > 0.99) in both analyses. Detailed results for Bland–Altman, Pearson correlation and intraclass correlation reliability can be found in Additional file 1: Table S2–S3, Fig. S4. Representative PET/CT images of CT-AC, PET and fused PET/CT are shown in Additional file 1: Fig. S5.

## Discussion

In the present study, both ZTE–AC and atlas-AC showed a good performance against CT-AC.

Recently, Schramm and Ladefoged described the current state of the art in metal artefact correction strategies for PET/MRI [31]. Several studies have investigated AC methods in the presence of dental implants [32–35], whereas only one has focused on MR-AC in postoperative patients with brain tumour and surgical metal implants [11].

Previous studies in the field investigated ZTE–AC and atlas-AC compared to gold standard ^68^GE transmission scan and reference standard CT-AC [10, 22, 36]. Those studies demonstrated a better performance of ZTE–AC when compared to the reference standard used in the study.

In the present study, no consistent superiority between the ZTE–AC and atlas-AC was found for brain PET/MRI AC in the hotspot region and postoperative site. Both methods presented mean bias within ± 5% across the regions, as previously described in a multi-centre study for MR-AC in brain PET/MRI [37]. Our findings are in line with a previous work comparing ZTE–AC and atlas-AC using brain ^15^O-labeled water (H_2_^15^O) PET/MRI [38]. The study showed no significant differences between AC for the regional values for cerebral blood flow. Notably, while the measurement of tracer uptake using H_2_^15^O PET/MRI is affected by small variations of cerebral blood flow, semi-quantitative measurement using MET PET may be robust enough to achieve clinical objectives. Part of the design of the present work differs from previously published studies regarding the selection of reference method [10], patient selection [36–39] and lack of metal implants [10, 22, 36, 38, 39].

In the present work, a 2D elliptical ROI was used for measurement of SUR values. Notably, previous works have used a volume of interest (VOI) for delineation [36]. Takano et al. [40] recently described the value of both 2D and 3D ROI in PET analyses showing a better performance for 2D ROIs in FDG PET, apparent diffusion coefficient (ADC) map, FA (fractional anisotropy), and a comparable p value for 2D ROIs and 3D ROIs (p value = 0.0056; p value = 0.0050, respectively) in MET PET. Based on these results, and for practical clinical usefulness in the radiological work-flow, a 2D ROI was chosen.

A limitation of this study is attributed to the relatively small number of patients included. This is explained by the high cost, limited use of PET/MRI, and the lack of universal validation in neuro-oncology [41].

Although the timing of PET/MRI was not standardised, the comparison between the AC methods might not be affected by the average post-injection time, since the acquisition for all the three AC maps was simultaneous. The aim of this study was not to render a diagnosis based on SUV values, which depend on acquisition timing, noise, tracers and tissue composition [42–44]. The evaluation of ZTE- and atlas-AC compared to CT-AC was performed on a patient-by-patient basis using SUV/SUR values, as previously reported in literature [11]. Further investigation will be necessary to explore the impact of the intrinsic differences between the AC maps on the accuracy of each AC-method compared to the reference gold standard, and to assess the severity of artefacts on MR-AC based on the size and appearance of the metal implant.

This is a single-centre, retrospective study. Future multi-centre prospective studies with larger cohorts, analysis of MRI biomarkers, single pathology brain tumours at the same treatment stage after diagnosis and analysis of MRI biomarkers will be needed to confirm our preliminary results. The study was performed using a single-reader assessment. Multi-reader assessment can be used in the upcoming study for ROI delineation, SUR analyses and inter-reader reliability. The study included all available patients from one institution during a specific time. Due to the retrospective nature of the study, no pre-study calculation of power was performed.

## Conclusions

This study retrospectively evaluated 14 pre- and postoperative patients with suspected or confirmed brain tumour. Good performance of ZTE–AC and atlas-AC compared to reference standard CT-AC was found for all analyses. Our results indicate that both ZTE and atlas are feasible MET PET/MRI AC methods.

## Supplementary information

**Additional file 1:** PDF file containing Supplementary material. Table S1. Hotspot analysis. Correlation and agreement for hotspot analysis in the overall group, metal and non-metal subgroups. As background, contralateral frontal cortex and mirror ROI were respectively assessed. ZTE/Atlas-AC compared to reference gold standard CT-AC. Table S2. Metal analysis. Correlation and agreement for parenchyma analysis in the metal subgroup. As background, contralateral mirror parenchyma ROI was assessed. ZTE/Atlas-AC compared to reference gold standard CT-AC. Table S3. Intraclass correlation reliability. Intraclass correlation reliability for absolute agreement in hotspot and metal analysis. Comparison among ZTE, Atlas and CT for SUR values. Fig. S1. Pearson correlation for hotspot analysis in the overall group. Correlation analysis for hotspot analysis in the overall group. As background, contralateral frontal cortex and mirror for maxSUR (a,b) and meanSUR (c,d) ROI were respectively assessed. ZTE/Atlas-AC compared to reference gold standard CT-AC. Fig. S2. Pearson correlation for hotspot analysis in the metal subgroup. Correlation analysis for hotspot analysis in the metal subgroup. As background, contralateral frontal cortex and mirror for maxSUR (a,b) and meanSUR (c,d) ROI were respectively assessed. ZTE/Atlas-AC compared to reference gold standard CT-AC. Fig. S3. Pearson correlation for hotspot analysis in the non-metal subgroup. Correlation analysis for hotspot analysis in the non-metal subgroup. As background, contralateral frontal cortex and mirror for maxSUR (a,b) and meanSUR (c,d) ROI were respectively assessed. ZTE/Atlas-AC compared to reference gold standard CT-AC. Fig. S4. Pearson correlation for metal analysis. Correlation for parenchyma analysis in the metal subgroup. As background, contralateral mirror parenchyma for maxSUR (a) and meanSUR (b) ROI was assessed. ZTE/Atlas-AC compared to reference gold standard CT-AC. Fig. S5. PET/CT images. Representative PET/CT images of CT-AC, PET and fused PET/CT are shown for a postoperative patient with metal implants.

## Data Availability

All data used and/or analysed during the current study are available from the corresponding author, Francesca De Luca (francesca.de.luca@ki.se), on reasonable request.

## Abbreviations

3D: Three-dimensional
AC: Attenuation correction
AW server: Advantage workstation server
CI: Confidence intervals
CT: Computer tomography
FET: Fluoroethyl-l-tyrosine
FDG: Flurodeoxyglucose
FOV: Field-of-view
FSPGR: Fast spoiled gradient echo
GE: General electric
H_2_^15^O: ^15^O-labeled water
ID: Identity
LAVA: Liver acquisition with volume acceleration
MET: Methionine
MR: Magnetic resonance
MRI: Magnetic resonance imaging
NEX: Number of excitations
OSEM: Ordered subset expectation maximization
PET: Positron emitting tomography
ROI: Region of interest
ROUT: Robust regression and outlier removal
SD: Standard deviation
SUR: Standardised uptake value ratio
SUV: Standardised uptake value
TOF: Time-of-flight
UTE: Ultrashort echo time
ZTE: Zero echo time
