# Supplementary material for: Validation of PET/MRI attenuation correction methodology in the study of brain tumours
Source: BMC Med Imaging. 2020 Nov 25;20:126. doi: 10.1186/s12880-020-00526-8 (PMC7690209; doi:10.1186/s12880-020-00526-8)

Validation of PET/MRI attenuation correction methodology in the study of brain tumours

Francesca De Luca [1,2*], Martin Bolin [1,3], Lennart Blomqvist [3,4] , Cecilia Wassberg [4], Heather Martin [2], Anna Falk Delgado [1,2]

[1] Department of Clinical Neuroscience, Karolinska Institutet, Stockholm, Sweden

[2] Department of Neuroradiology, Karolinska University Hospital, Stockholm, Sweden

[3] Department of Molecular Medicine and Surgery, Karolinska Institutet, Stockholm, Sweden

[4] Department of Medical Radiation Physics and Nuclear Medicine, Karolinska University Hospital, Stockholm, Sweden

Supplementary material

Table S1. Hotspot analysis. Correlation and agreement for hotspot analysis in the overall group, metal and non-metal subgroups. As background, contralateral frontal cortex and mirror ROI were respectively assessed. ZTE/Atlas-AC compared to reference gold standard CT-AC.

Group	Hotspot analysis	MR-AC	ρ	bias	SD	95% CI from	95% CI to
**Overall**		**ZTE vs CT**					
	maxSURhotspot/cortex		0.9955	0.05	2.84	-1.41	1.51
	meanSURhotspot/cortex		0.9969	-0.02	2.54	-1.32	1.28
	maxSURhotspot/mirror		0.9994	-0.02	1.04	-0.55	0.51
	meanSURhotspot/mirror		0.9991	0.82	1.72	-0.07	1.70
**Overall**		**ATLAS vs CT**					
	maxSURhotspot/cortex		0.9901	1.29	4.56	-1.05	3.64
	meanSURhotspot/cortex		0.9926	1.62	4.31	-0.60	3.83
	maxSURhotspot/mirror		0.9973	0.10	2.69	-1.28	1.48
	meanSURhotspot/mirror		0.9947	1.21	3.25	-0.46	2.88
**Metal**		**ZTE vs CT**					
	maxSURhotspot/cortex		0.9963	-1.62	1.50	-3.00	-0.24
	meanSURhotspot/cortex		0.9982	-1.80	1.24	-2.95	-0.66
	maxSURhotspot/mirror		0.9974	-0.40	1.30	-1.60	0.81
	meanSURhotspot/mirror		0.9988	0.66	1.67	-0.88	2.20
**Metal**		**ATLAS vs CT**					
	maxSURhotspot/cortex		0.9876	-0.23	3.87	-3.81	3.35
	meanSURhotspot/cortex		0.9933	0.54	3.48	-2.68	3.76
	maxSURhotspot/mirror		0.9912	0.28	3.35	-2.82	3.38
	meanSURhotspot/mirror		0.9965	1.63	3.25	-1.38	4.64
**Non-metal**		**ZTE vs CT**					
	maxSURhotspot/cortex		0.9965	1.21	3.04	-0.96	3.38
	meanSURhotspot/cortex		0.9978	1.23	2.49	-0.55	3.01
	maxSURhotspot/mirror		0.9997	0.24	0.77	-0.31	0.79
	meanSURhotspot/mirror		0.9992	0.92	1.83	-0.39	2.24
**Non-metal**		**ATLAS vs CT**					
	maxSURhotspot/cortex		0.993	2.36	4.90	-1.14	5.86
	meanSURhotspot/cortex		0.9938	2.37	4.83	-1.08	5.83
	maxSURhotspot/mirror		0.9981	-0.03	2.30	-1.68	1.62
	meanSURhotspot/mirror		0.9942	0.92	3.39	-1.50	3.35
Pearson correlation, pvalue< 0.0001							
ρ: Pearson correlation; bias: Bland-Altman; SD: standard deviation; CI: confidence intervals							

Table S2. Metal analysis. Correlation and agreement for parenchyma analysis in the metal subgroup. As background, contralateral mirror parenchyma ROI was assessed. ZTE/Atlas-AC compared to reference gold standard CT-AC.

Group	Metal analysis	MR-AC	ρ	bias	SD	95% CI from	95% CI to
**Metal**		**ZTE vs CT**					
	maxSURmetal/mirror		0.9940	0.21	2.28	-1.70	2.11
	meanSURmetal/mirror		0.9938	0.48	2.50	-1.61	2.57
**Metal**		**ATLAS vs CT**					
	maxSURmetal/mirror		0.9929	1.54	4.01	-1.81	4.89
	meanSURmetal/mirror		0.9885	0.56	3.64	-2.48	3.61
Pearson correlation, pvalue< 0.0001							
ρ: Pearson correlation; bias: Bland-Altman; SD: standard deviation; CI: confidence intervals							

Table S3. Intraclass correlation reliability. Intraclass correlation reliability for absolute agreement in hotspot and metal analysis. Comparison among ZTE, Atlas and CT for SUR values.

Group	Hotspot analysis	ICC
**Overall**		
	maxSURhotspot/cortex	0.993
	meanSURhotspot/cortex	0.995
	maxSURhotspot/mirror	0.998
	meanSURhotspot/mirror	0.996
**Metal**		
	maxSURhotspot/cortex	0.990
	meanSURhotspot/cortex	0.992
	maxSURhotspot/mirror	0.994
	meanSURhotspot/mirror	0.996
**Non-metal**		
	maxSURhotspot/cortex	0.994
	meanSURhotspot/cortex	0.996
	maxSURhotspot/mirror	0.998
	meanSURhotspot/mirror	0.996
	**Metal analysis**	
**Metal**		
	maxSURmetal/mirror	0.990
	meanSURmetal/mirror	0.992

Fig. S1. Pearson correlation for hotspot analysis in the overall group. Correlation analysis for hotspot analysis in the overall group. As background, contralateral frontal cortex and mirror for maxSUR (a,b) and meanSUR (c,d) ROI were respectively assessed. ZTE/Atlas-AC compared to reference gold standard CT-AC.

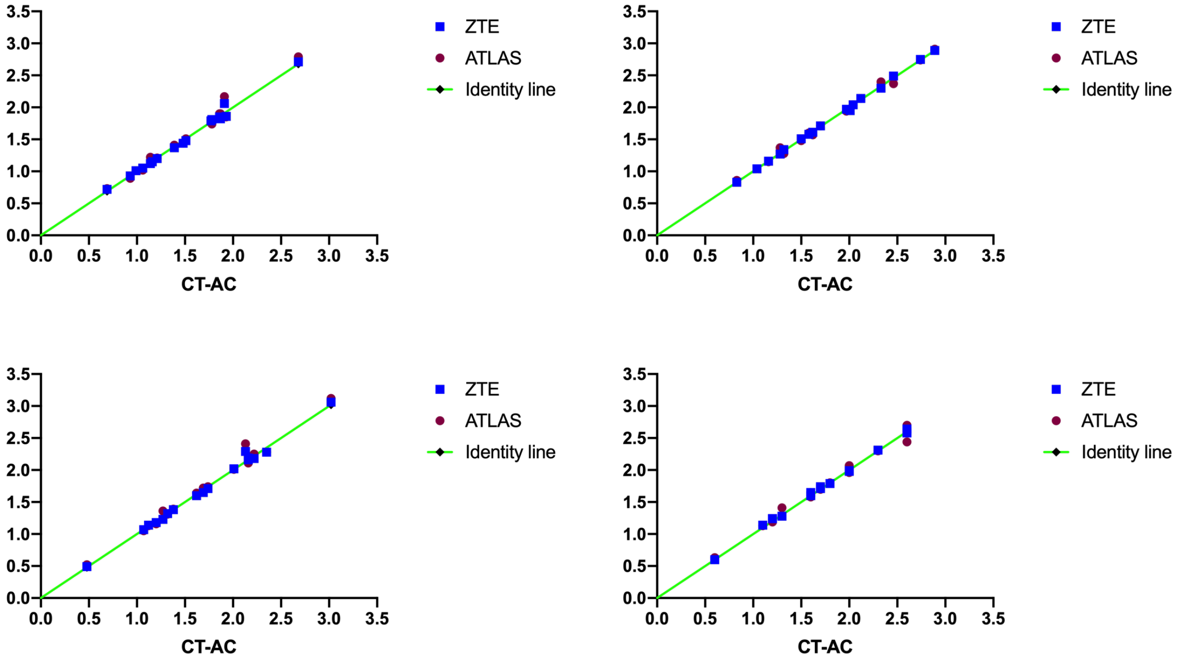

Fig. S2. Pearson correlation for hotspot analysis in the metal subgroup. Correlation analysis for hotspot analysis in the metal subgroup. As background, contralateral frontal cortex and mirror for maxSUR (a,b) and meanSUR (c,d) ROI were respectively assessed. ZTE/Atlas-AC compared to reference gold standard CT-AC.

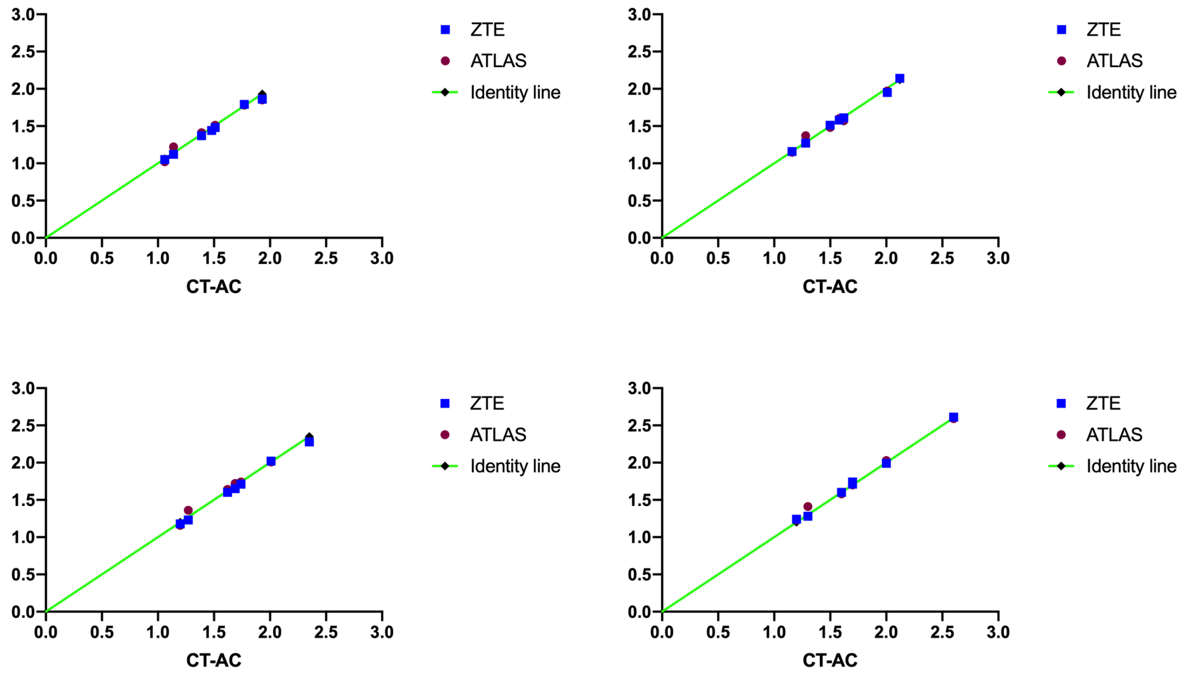

Fig. S3. Pearson correlation for hotspot analysis in the non-metal subgroup. Correlation analysis for hotspot analysis in the non-metal subgroup. As background, contralateral frontal cortex and mirror for maxSUR (a,b) and meanSUR (c,d) ROI were respectively assessed. ZTE/Atlas-AC compared to reference gold standard CT-AC.

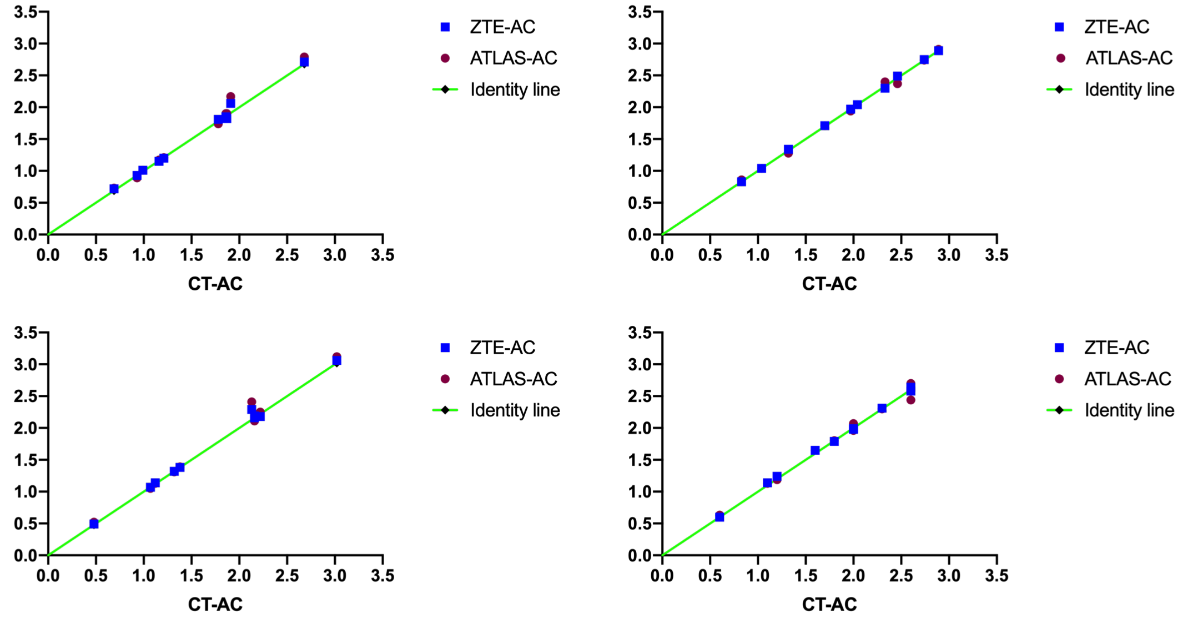

Fig. S4. Pearson correlation for metal analysis. Correlation for parenchyma analysis in the metal subgroup. As background, contralateral mirror parenchyma for maxSUR (a) and meanSUR (b) ROI was assessed. ZTE/Atlas-AC compared to reference gold standard CT-AC.

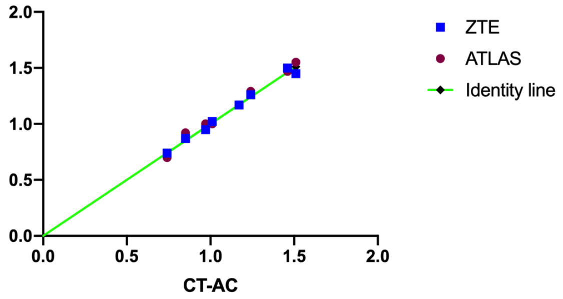

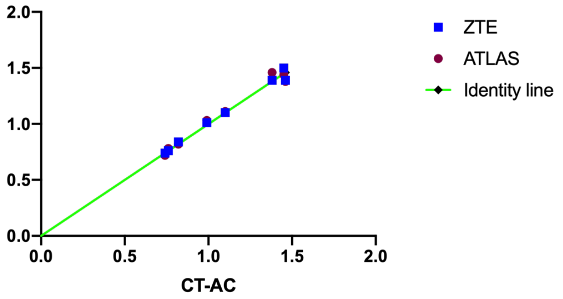

Fig. S5. PET/CT images. Representative PET/CT images of CT-AC, PET and fused PET/CT are shown for a postoperative patient with metal implants.

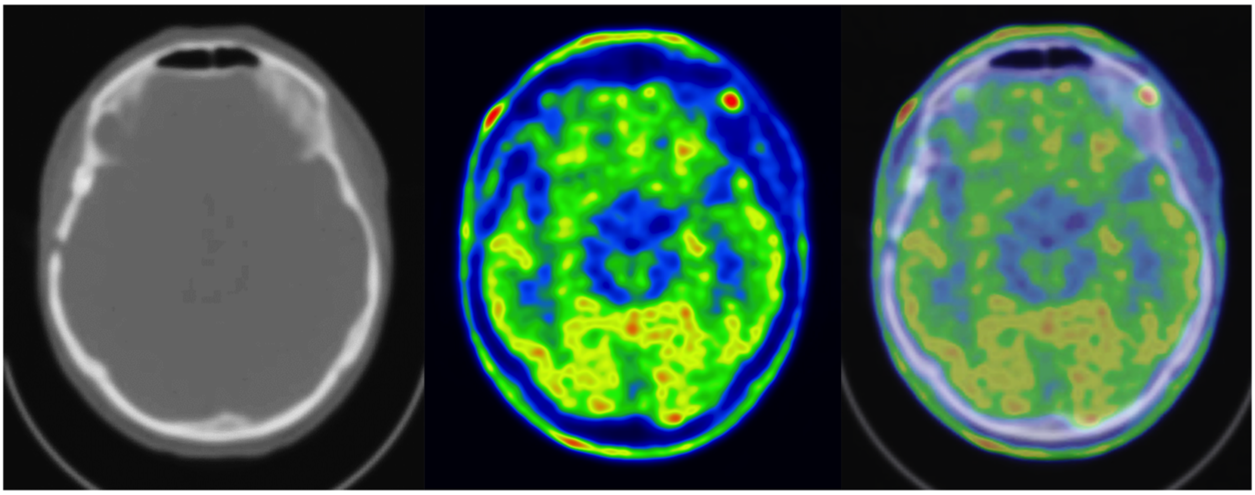

Supplement: Supplementary file 1 — Additional file 1: PDF file containing Supplementary material. Table S1. Hotspot analysis. Correlation and agreement for hotspot analysis in the overall group, metal and non-metal subgroups. As background, contralateral frontal cortex and mirror ROI were respectively assessed. ZTE/Atlas-AC compared to reference gold standard CT-AC. Table S2. Metal analysis. Correlation and agreement for parenchyma analysis in the metal subgroup. As background, contralateral mirror parenchyma ROI was assessed. ZTE/Atlas-AC compared to reference gold standard CT-AC. Table S3. Intraclass correlation reliability. Intraclass correlation reliability for absolute agreement in hotspot and metal analysis. Comparison among ZTE, Atlas and CT for SUR values. Fig. S1. Pearson correlation for hotspot analysis in the overall group. Correlation analysis for hotspot analysis in the overall group. As background, contralateral frontal cortex and mirror for maxSUR (a,b) and meanSUR (c,d) ROI were respectively assessed. ZTE/Atlas-AC compared to reference gold standard CT-AC. Fig. S2. Pearson correlation for hotspot analysis in the metal subgroup. Correlation analysis for hotspot analysis in the metal subgroup. As background, contralateral frontal cortex and mirror for maxSUR (a,b) and meanSUR (c,d) ROI were respectively assessed. ZTE/Atlas-AC compared to reference gold standard CT-AC. Fig. S3. Pearson correlation for hotspot analysis in the non-metal subgroup. Correlation analysis for hotspot analysis in the non-metal subgroup. As background, contralateral frontal cortex and mirror for maxSUR (a,b) and meanSUR (c,d) ROI were respectively assessed. ZTE/Atlas-AC compared to reference gold standard CT-AC. Fig. S4. Pearson correlation for metal analysis. Correlation for parenchyma analysis in the metal subgroup. As background, contralateral mirror parenchyma for maxSUR (a) and meanSUR (b) ROI was assessed. ZTE/Atlas-AC compared to reference gold standard CT-AC. Fig. S5. PET/CT images. Representative PET/CT [file 12880_2020_526_MOESM1_ESM.pdf]
